# Mepivacaine-induced anaphylactic shock in a pregnant woman undergoing combined spinal and epidural anesthesia for cesarean delivery: a case report

**DOI:** 10.1186/s40981-019-0302-6

**Published:** 2019-12-19

**Authors:** Miyuki Takahashi, Kunihisa Hotta, Soichiro Inoue, Tomonori Takazawa, Tatsuo Horiuchi, Takashi Igarashi, Mamoru Takeuchi

**Affiliations:** 1 Department of Anesthesiology and Critical Care Medicine, Jichi Medical University, 3311-1 Yakushiji, Shimotsuke-shi, Tochigi, 329-0498 Japan; 2 Department of Anesthesiology, St. Marianna University School of Medicine, 2-16-1 Sugao, Miyamae-ku, Kawasak-shii, Kanagawa 216-8511 Japan; 3 Intensive Care Unit, Gunma University Hospital, 3-39-15 Showa-machi, Maebashi-shi, Gunma 371-8511 Japan; 4 Department of Anesthesiology, Gunma University Graduate School of Medicine, 3-39-22 Showa-machi, Maebashi-shi, Gunma 371-8511 Japan

**Keywords:** Anaphylaxis, Pregnancy, Cesarean delivery, Spinal anesthesia, Local anesthetic, Mepivacaine, Basophil activation test

## Abstract

**Background:**

Anaphylactic shock during pregnancy is a rare but life-threatening event for both the mother and the newborn.

**Case presentation:**

A 42-year-old woman, who was pregnant with twins, was scheduled for cesarean delivery under combined spinal and epidural anesthesia. An epidural catheter was placed uneventfully. After spinal anesthesia, the patient exhibited skin symptoms and severe hypotension. The patient was diagnosed with anaphylaxis, and subsequently, treatment was started. Fetal heart rate monitoring revealed sustained bradycardia, and it was decided to proceed with cesarean delivery. After delivery, the mother’s vital signs recovered. Both infants were intubated due to birth asphyxia. Currently, the twins are 4 years old and exhibit no developmental problems. Clinical examination identified mepivacaine as the causative agent of anaphylaxis.

**Conclusions:**

This case report highlights that upon occurrence of anaphylaxis during pregnancy, maternal treatment and fetal assessment should be started immediately. Indication for immediate cesarean delivery should be considered and a definite identification of the causative factor pursued.

## Background

Anaphylactic shock during pregnancy is a rare but life-threatening event for both the mother and the newborn. The estimated incidence is 1.6 to 3.8 cases per 100,000 maternities, which is much lower than that in other populations [1–3]. Since numerous drugs are contraindicated for pregnant women, the fewer opportunities for drug use may contribute to the low incidence of anaphylaxis during pregnancy. Antibiotics have been reported as the main causative agent of anaphylaxis during pregnancy, whereas local anesthetics have rarely been reported [1, 2].

Managing anaphylactic shock during pregnancy is challenging, since the fetus’ condition and how it is affected by treatment must be considered. Upon occurrence of anaphylaxis during late pregnancy, the optimal timing for delivery and anesthetic management remain controversial. Emergency cesarean delivery may benefit the newborn but poses a risk for the mother, whose condition may be unstable [4]. Each patient’s clinical treatment strategy should therefore be determined based on anaphylaxis severity, maternal condition, and fetal status.

Here, we report a case of anaphylaxis in a pregnant woman undergoing combined spinal and epidural anesthesia for cesarean delivery. Clinical examination identified mepivacaine as the causative agent.

## Case presentation

A 42-year-old woman, who was pregnant with twins, was scheduled for cesarean section at 37 weeks of gestation under combined spinal and epidural anesthesia. The woman had atopic dermatitis but no past history of drug allergy. After arrival at the operating room, intravenous administration of hydroxyethylated starch was started. Combined spinal and epidural anesthesia was administered in the right lateral position, at the L3–L4 and the Th12–L1 interspaces, respectively. After local infiltration of 6 mL of preservative-free 1% mepivacaine, the epidural space was identified by loss of resistance to saline, and an epidural catheter was placed. An aspiration test was confirmed as negative, and a test dose of 1% mepivacaine (3 ml) was administered via the catheter. During spinal anesthesia, maternal blood pressure became unmeasurable with a noninvasive blood pressure monitor. Lumbar puncture was successfully performed, and 10 mg of 0.5% hyperbaric bupivacaine and 20 μg of fentanyl were intrathecally administered. After returning the patient to the supine position, her face was swollen, and she exhibited erythema all over the body. Maternal blood pressure and heart rate were 74/56 mmHg and 112 beats/min, respectively. The mother was diagnosed with anaphylaxis and immediately received infusion of Ringer’s solution with left uterine displacement. Intravenous phenylephrine was intermittently administered. Despite hemodynamic instability, the mother’s breathing remained stable at 98% of oxygen saturation on room air, and oxygen was administered via a face mask. Fetal heart rate monitoring revealed sustained fetal bradycardia of 80 beats/min. Maternal systolic blood pressure remained around 80–90 mmHg under repeated administration of phenylephrine and transfusion. Fetal bradycardia was not recovered. After confirming the sensory block level of Th4, it was decided to proceed with cesarean delivery. The infants were delivered 17 and 18 min after anaphylaxis onset, both without spontaneous respiration, and were intubated and transferred to the neonatal intensive care unit. At 1 and 5 min, the apgar scores were 2 and 4 for the first infant and 2 and 5 for the second infant, respectively. Analysis of the umbilical artery blood revealed a pH of 6.842 for the first infant and 6.775 for the second infant. After delivery, the mother’s vital signs were recovered and remained stable. A two-phase allergic reaction was prevented through administration of 500 mg of methylprednisolone. After surgery, the mother was continuously monitored in the maternity ward, and her clinical course remained uneventful, being discharged 6 days after surgery. The first infant was extubated 2 days after birth and discharged 13 days after birth, whereas the second infant required further examination and treatment after having seizures. He was discharged with an oral anticonvulsant 16 days after birth, after which he remained seizure-free. The anticonvulsant was discontinued at 6 months of age. Currently, the twins are 4 years old and exhibit no developmental problems.

**Fig. 1:**
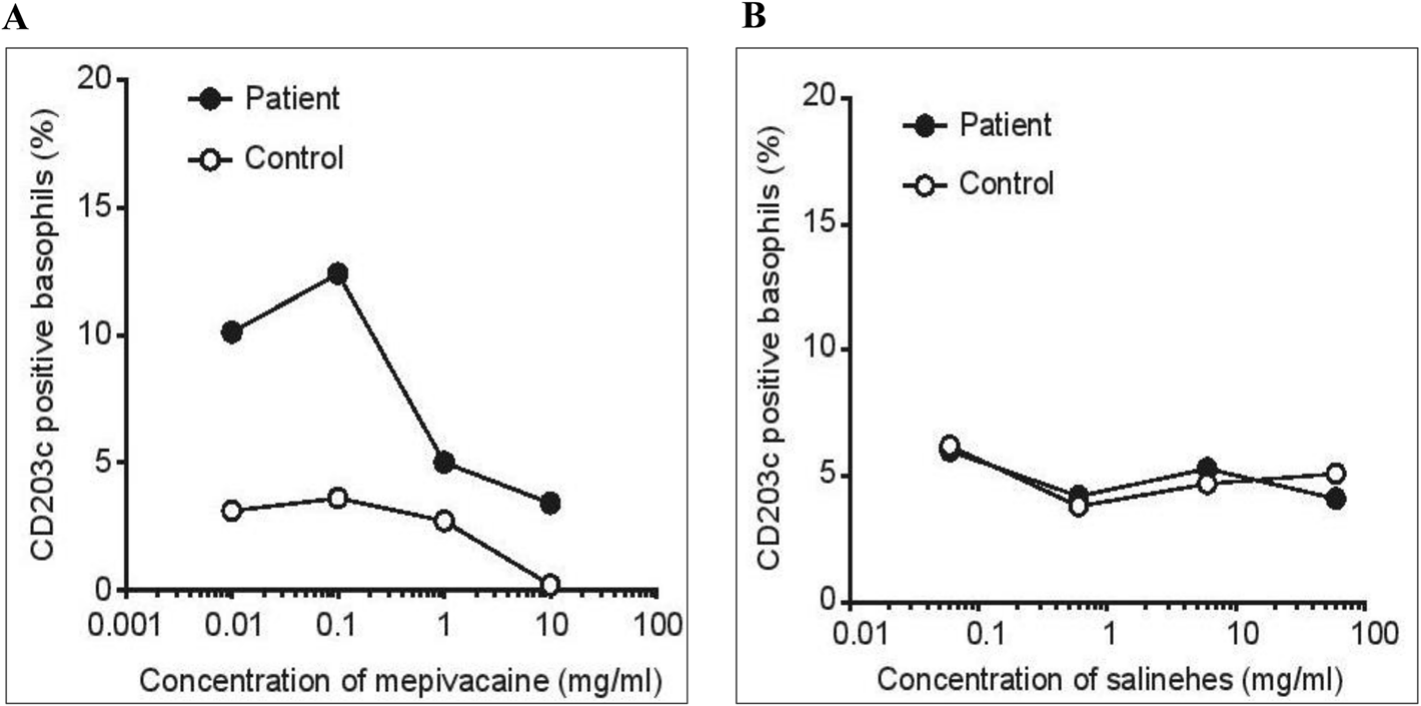
Basophil activation test. Flow cytometric analysis of basophils expressing CD203c. **a** The patient’s mepivacaine-induced CD203c upregulation. **b** Hydroxyethylated starch did not induce CD203c upregulation

**Fig. 2:**
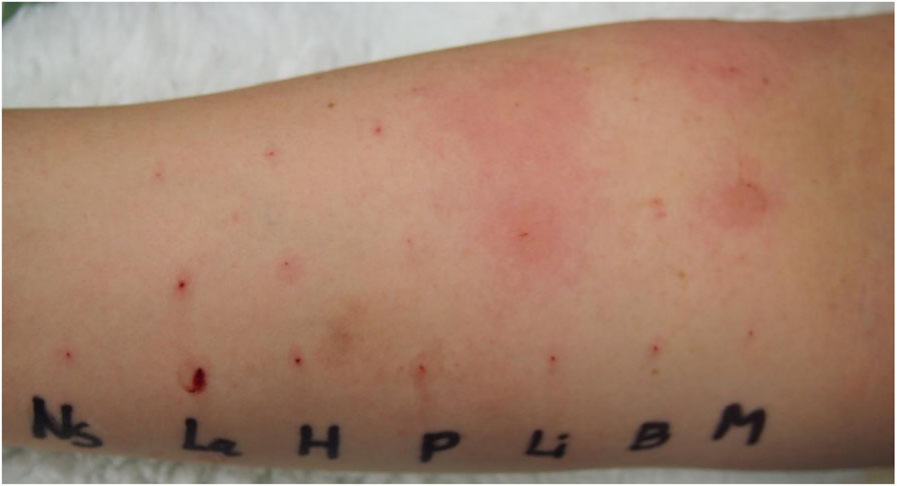
Skin prick test. NS saline, La latex, H hydroxyethylated starch, P procaine, L lidocaine, B bupivacaine, M mepivacaine. Positive reaction to lidocaine and mepivacaine

We suggested the patient to be examined to determine the causative agent of anaphylaxis. The patient agreed to be subjected to the basophil activation test (BAT), but not the skin test. The BAT was performed 9 months after the operation for mepivacaine and hydroxyethylated starch, providing a positive result to mepivacaine (Fig 1). The patient was then suggested once more to undergo the skin test to confirm the accuracy of the BAT results and to investigate cross-reactivity with other local anesthetics. The skin prick test was performed 10 months after the operation, for latex, hydroxyethylated starch, procaine, lidocaine, bupivacaine, and mepivacaine, providing a positive reaction to both lidocaine and mepivacaine (Fig 2).

## Discussion

The present case describes anaphylaxis in a pregnant woman who underwent combined spinal and epidural anesthesia for cesarean delivery. The immediate diagnosis, treatment, and cesarean delivery may have led to the good outcomes achieved for both the mother and the newborns.

When treating anaphylaxis during late pregnancy, besides comprehensive treatment, care should be taken to evaluate the fetal condition and to decide the best delivery timing. Primary management of anaphylaxis includes immediate withdrawal of antigen administration, seeking help, airway maintenance, aggressive fluid resuscitation, and adrenaline administration. Examining serum tryptase and histamine levels is useful for diagnosing anaphylaxis. Additionally, obstetric management may involve left uterine displacement and fetal monitoring. Maternal hypotension immediately affects the placental circulation and fetal status. However, the American College of Obstetricians and Gynecologists indicates that, during anaphylaxis, a stable maternal hemodynamic status does not ensure adequate placental perfusion and fetal oxygenation, whereas normal fetal heart rate variability reassures fetal status [5]. Previous studies recommend that emergency cesarean delivery should be considered in cases of persistent maternal hemodynamic instability, despite resuscitation [4, 6]. In the present case, monitoring fetal heart rate was the key for the decision to perform a cesarean delivery. Although the good outcomes were achieved, fetal bradycardia was not recovered by phenylephrine. Administration of adrenaline for anaphylaxis may have increased cardiac output and systemic vascular resistance, resulting in improved uteroplacental perfusion [7].

Among previous case reports of anaphylactic labor, 46% resulted in adverse fetal neurologic outcomes [8–13]. In such cases, the anaphylactic parturients either had a delayed cesarean delivery [8, 9, 13] or did not receive sufficient adrenaline to manage severe hypotension [11]. The fetal neurologic outcome was much better in cases of anaphylaxis occurring during cesarean delivery, which can be attributed to a short duration of fetal cerebral ischemia [4]. The benefits of emergency cesarean delivery for anaphylactic patients refractory to medical treatment need to be balanced against the risks of surgery in pregnant women with an unstable general condition. If the time of gestation is less than 32 weeks, the risks of neonatal morbidity and mortality should also be considered. Anaphylaxis-related cardiovascular disturbance can be enhanced in pregnant patients by inferior vena cava compression. Moreover, neuraxial anesthesia blocks the sympathetic nerve, often causing hypotension. For anaphylaxis occurring during cesarean delivery, maternal morbidity due to severe complications was reported in 20% of cases [4]. In the present case, anaphylaxis occurred during the intrapartum period, and the immediate cesarean delivery exerted a beneficial impact both on the mother and the infants.

Identifying the causative agent of anaphylaxis is essential to prevent allergic reactions. Agents, or known as allergens, administered immediately before an event, are often determined as culprit allergens without subsequent examinations. Had we not pursued the cause of the allergic reaction, we might have mistakenly assumed that the hydroxyethylated starch was the allergen, since anesthetic allergies have rarely been reported. Therefore, it should be kept in mind that the suspected agent may not be the true causative agent, and incorrect speculation may place the patient at risk of further exposure to the true allergen or cause unnecessary avoidance of harmless effective drugs.

The skin prick test is the gold standard to determine the cause of anaphylaxis, although it carries the risk of immediate hypersensitivity reactions. On the other hand, the BAT is an in vitro examination, which poses no risk of anaphylactic reactions. The BAT is based on the upregulation of granule-derived markers expressed at the basophil membrane upon ex vivo activation by the suspected agent. It recently became widely accepted as an additional and reliable tool, with high sensitivity and specificity to identify the causative agent of immediate drug hypersensitivity [14–17]. Further studies are required to evaluate the usefulness of this tool as a diagnostic approach of anaphylaxis, although diagnostic precision can be improved by combining multiple methods, such as the skin test and the BAT. In the present case, positive reactions to mepivacaine in both the BAT and skin prick test would support the reliability of BAT as a diagnostic tool.

In summary, the present case highlights that, upon anaphylaxis during pregnancy, maternal treatment and fetal heart rate monitoring should be started immediately. If the maternal hemodynamic status does not recover or if persistent non-reassuring fetal heart rate patterns are observed, immediate cesarean delivery should be considered, especially at the intrapartum period. Moreover, pursuing a definite diagnosis of the culprit allergen is beneficial for patients to prevent allergic reactions.

## Data Availability

Not applicable

## Abbreviations

BAT: Basophil activation test
